# Systematic Identification of Machine-Learning Models Aimed to Classify Critical Residues for Protein Function from Protein Structure

**DOI:** 10.3390/molecules22101673

**Published:** 2017-10-09

**Authors:** Ricardo Corral-Corral, Jesús A. Beltrán, Carlos A. Brizuela, Gabriel Del Rio

**Affiliations:** 1 Department of Biochemistry and Structural Biology, Instituto de Fisiologa Celular, Universidad Nacional Autónoma de México, México D.F. 04510, Mexico; rcorral@email.ifc.unam.mx; 2 Computer Science Department, CICESE Research Center, Ensenada, Baja California 22860, Mexico; abeltran@cicese.edu.mx (J.A.B.); cbrizuel@cicese.mx (C.A.B.)

**Keywords:** protein structure, functional residues, machine learning

## Abstract

Protein structure and protein function should be related, yet the nature of this relationship remains unsolved. Mapping the critical residues for protein function with protein structure features represents an opportunity to explore this relationship, yet two important limitations have precluded a proper analysis of the structure-function relationship of proteins: (i) the lack of a formal definition of what critical residues are and (ii) the lack of a systematic evaluation of methods and protein structure features. To address this problem, here we introduce an index to quantify the protein-function criticality of a residue based on experimental data and a strategy aimed to optimize both, descriptors of protein structure (physicochemical and centrality descriptors) and machine learning algorithms, to minimize the error in the classification of critical residues. We observed that both physicochemical and centrality descriptors of residues effectively relate protein structure and protein function, and that physicochemical descriptors better describe critical residues. We also show that critical residues are better classified when residue criticality is considered as a binary attribute (i.e., residues are considered critical or not critical). Using this binary annotation for critical residues 8 models rendered accurate and non-overlapping classification of critical residues, confirming the multi-factorial character of the structure-function relationship of proteins.

## 1. Introduction

The study of the relationship between protein structure and protein function constitutes an open problem in biochemistry and bioinformatics. The main motivation for addressing this problem is dual: on the one hand it may facilitate the annotation of protein function and on the other hand it may help understand the mechanism(s) by which a given protein structure renders a particular function. Given the accumulation of protein sequences most efforts to predict protein function are based on this information. Alternatively, the mechanisms of protein function are studied based on the atomic three-dimensional structure of proteins (hereafter referred to as protein structure). Despite this difference in the data source, these two approaches converge in the prediction of critical residues for protein function revealing the relevance of this subject [1]. Critical residues may be ones critical for protein stability, folding, binding and/or catalysis performed by the protein of interest, hence these residues hold key information to understand the structure-function relationship of proteins. While mutations may reduce or improve protein function, in this work we will refer to critical residues as those that serve to maintain the function of proteins, hence mutations on these residues may affect negatively the protein function. There are different reports aimed to predict active site residues [2,3,4], binding site residues [5,6,7,8,9] or stability and/or folding residues [10,11,12,13], yet few works have been reported to computationally identify critical residues [14,15,16]. From these studies it is important to note the lack of a quantitative definition of critical residues for protein function that serves to effectively train machine-learning approaches based on mutagenesis data. The lack of a formal definition of what a critical residue for protein function is represents an important limitation in the field to properly model this class of residues. It is our goal in this work to contribute in the definition of such class of protein’s residues.

We will focus on mapping the protein function to its structure instead onto its sequence considering the degeneracy of this relationship. That is, since protein sequence-structure relationship is degenerated (i.e., many different protein sequences may render very similar protein structure) and protein structure-function relationship is degenerated as well (i.e., different protein structures may render very similar protein function), by mapping the protein structure-function relationship we aim to model only one level of degeneracy in this relationship. Furthermore, considering the multiple roles (e.g., stability, folding) critical residues play in protein function, it is expected that different attributes of protein structure and multiple algorithms may be required to predict these residues; in such case, it may be anticipated the need to systematically test each of these factors in the structure-function relationship of proteins. Hence, it is desirable to analyze the structure-function relationship of proteins mathematically. For instance, lets define a protein structure *p*_i_ as part of the set of protein structures ***P*** and a protein function *a*_i_ as part of the set of protein functions ***A***. Protein structure is defined in turn by two sets: the group of amino acid residues an *i*-protein is made of (***AA***_i_) and the group of relationships between these residues within the *i*-protein (***E***_i_). Alternatively, protein function may be represented by the set of critical residues (***CR***). Hence, ***P*** may be related to ***A*** through these three sets (***AA***, ***E*** and ***CR***). We have previously noted that the relationship between the protein function and the protein structure may be modeled by a mathematical function of the bijection type [17]. Such condition is satisfied in proteins because there are no two proteins known with identical values for stability, folding rate, catalytic constants, etc, hence it is expected that each protein will display different sets of critical residues, yet with some overlaps (e.g., catalytic residues). Hence, the structure-function problem of proteins may be mathematically posed by the following expression (Equation (1)):

*f*(***P***) = ***A***; *f*(***AA***, ***E***) = ***CR*** (1)

Two problems can be recognized in this mathematical function: (i) the representation of ***P*** and ***A***, and (ii) to determine the form of the function. About this last problem, the function may be a bijection represented by multiple mathematical functions considering the multiple roles critical residues play in protein function, yet this has to be tested. In terms of protein structure representation, different approaches have been described over the past years. For instance, protein sequences may be represented by different physicochemical properties (e.g., hydrophobicity, hydrophilicity), sequence composition (e.g., number of different amino acids), among others, while protein structure may also include protein secondary structure (e.g., α-helical, β-sheet), amino-acids flexibility and amino-acids surface exposure, among others. A recent development allows researchers to calculate most of these descriptors using a single framework, ProtDCal (Protein Descriptors Calculation program, version 3.8, University of Waikato, Hamilton, New Zealand) [18]. Yet, descriptors based on sequence conservation based on protein structure or centrality measures shown to effectively classify critical residues for protein function are not included in ProtDCal.

We propose a procedure based on machine-learning algorithms to test which of these multiple representations of protein structure are useful to classify critical residues. Machine learning is an area of artificial intelligence that builds models from data [19], instead of fitting data to first-principle models. From the perspective of machine learning, the set of features for protein structure (derived from the set ***AA*** and ***E*** sets) is used to learn a function of ***P*** (*f*: ***P***- > ***A***) that renders the protein activity (***A***). Ideally, this target function *f*(***P***) is learnable, yet in practice it is not (i.e., lack of sufficient data among others). Instead, machine learning aims to identify an alternative function (Equation (2)) of ***P*** (*g*(***P***)) such that:

*g*(***P***) ≈ *f*(***P***); *g*(***P***) = ***A***; *g*(***AA***, ***E***) = ***CR*** (2)

In this work we developed a supervised learning setup, where examples in ***CR*** were taken from the literature.

Hence, machine-learning approaches constitute a tool that allow researcher to test both data-driven models and features that best describe observable phenomena. To facilitate the identification of the best model to describe the *g*(***P***) function, a combined selection of the largest data set of classification algorithms publicly available and parameter optimization has been implemented in AutoWEKA version 3.8, a suite of machine-learning algorithms coded in Java programming language [20]. This optimization in combination with the list of descriptors available nowadays of protein structure allows for the identification of the best model to classify critical residues for protein function. This effort is relevant considering that machine-learning algorithms have been designed to work with multiple features and the first models aimed to predict critical residues were reported using one or few features derived from protein sequence or protein structure. Yet, a recent report aimed to predict critical residues for protein function based on machine learning approaches [21] used only one algorithm and few descriptors were tested. 

Hence, the aim of this work is to test different descriptors of protein structure (ProtDCal and centralities) to identify the best machine-learning models to classify critical residues for protein function. To achieve this goal, here we report an index to quantify and/or label the relevance for protein function a residue has based on mutagenesis data. Our results show that both physicochemical and centrality descriptors effectively identify critical residues for protein function and that physicochemical descriptors are better descriptors of critical residues than centrality measures. We also show that critical residues are best predicted when a binary annotation (critical, non-critical) is assumed. We provide evidence that combining multiple models to relate protein structure and protein function correctly classify every known critical residue. Even though we define critical residues for specific proteins, in order for our model to learn “by example”, our procedure allow us to then generalize the concept for new proteins. The scenario will be as follows: the learning model will be given as input a set of molecular descriptors and the model will identify critical residues in this protein as long as these descriptors share, at the critical residues, some common pattern with the proteins used in the training process. Notice that as we enlarge the set of examples with diverse samples of proteins/functions then our learning model will improve. While the results presented in this work used a limited set of proteins and only globular proteins, the significance of our results improve state of the art predictions and the strategy presented here may be reused with new experimental data on critical residues, repeating the prediction-validation cycle that would lead to a robust prediction and better understanding of these residues (i.e., *g*(***P***) = *f*(***P***)).

## 2. Results

Our work aims to first test which descriptors of protein structure are relevant to classify critical residues for protein function; for this goal we used different definitions of critical residues for protein function previously reported in the literature. Alternatively, we aim to test if critical residues for protein function may be better modeled by a unique and quantitative index (CI index, see Methods). These aims allowed us to identify machine-learning models to classify critical residues for protein function; such models were used to explore the nature of structure-function relationship of proteins.

### 2.1. Data Sets and Protein Structure Descriptors Used to Train Machine-Learning Models to Classify Critical Residues for Protein Function

We created three sets of proteins for which the structure is known and experimental mutagenesis data are available. That is, all data sets used in this study were derived from experimental data; this restricts the number of proteins analyzed (11 globular proteins, see Methods), yet the number of residues considered in our study is sufficiently large (2129 residues) to perform statistical tests to define the confidence of our results. The first set was the one previously described by our group to predict critical residues for protein function based on centralities [16]; this set included 6 proteins that classified critical or not critical residues according to the different criterion reported by the authors mutating each of those 6 proteins and is referred to as JAMMING1SET (see Materials and Methods). The second data set, JAMMING2SET, included site-directed mutagenesis results reported for other five proteins and each position received a criticality score; here, the criticality score (*CI*) for each residue was computed based on the reported mutants (see description on the criticality index *CI* below). The third data set, JAMMING3SET, was derived from the JAMMING2SET where every residue was labeled as critical or not critical depending on a cutoff value of the criticality score (*CI* ≤ 0.5 for non-critical residues and *CI* > 0.5 for critical residues). For each data set, three sets of descriptors were used, including: (i) 2789 descriptors derived from ProtDCal; (ii) 11 centrality measures and one structure-based conservation score and (iii) the union set from the previous two sets (see Methods). Hence, nine data sets were used to identify the best algorithm and parameters to classify critical residues.

### 2.2. Testing Protein Structure Descriptors to Classify Critical Residues for Protein Function

**Figure 1:**
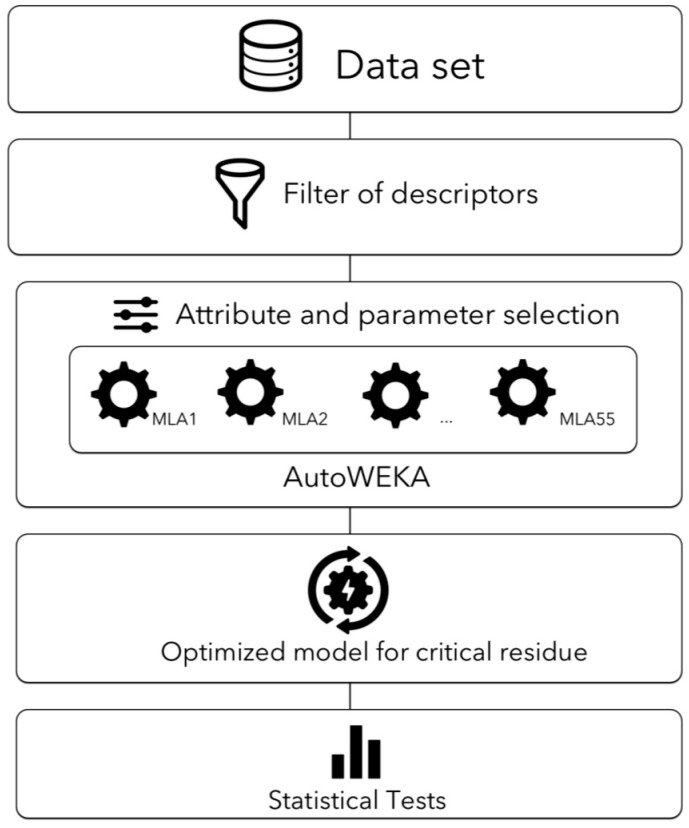
Diagram to identify optimized models to predict critical residues. The figure represents the data sets (in our study we used 3, but these can be modified as more information becomes available) and initial filtering operation to perform the optimization of attributes (2789 ProtDCal attributes plus 11 centralities), the machine-learning algorithm (MLA) and its corresponding parameters, to reduce the error in the classification of critical residues as performed by AutoWEKA. 55 MLA currently implemented in WEKA were used in this work. The optimized model then is tested by cross-validation using the training set (leave-one-out) or by swapping training sets as test sets. This strategy was implemented and executed using WEKA (see Methods for further details).

Our first step to identify the most appropriate descriptors and algorithm to classify critical residues was to filter out descriptors; this filtering eliminated all descriptors that did not change among critical and non-critical residues (see Methods). The second step aimed to identify the best machine-learning algorithm and its parameters to learn from that filtered data set using AutoWEKA. We report here two models obtained at two different execution times for each dataset: For each dataset we derived two best models trained with centralities, two with ProtDCal descriptors and two other with the union set of centralities and ProtDCal descriptors (see Materials and Methods). A third step was to test the different classifiers using the leave-one out procedure. Figure 1 summarizes our strategy. 

We chose different statistical parameters to evaluate the efficiency of the model to classify critical residues. In a first instance, we report the root average error (as reported by AutoWEKA) at finding the optimum algorithm-parameters-descriptors to classify critical residues (see Table 1, Table 4 and Table 6); this error estimates how learnable are the data used to classify critical residues. Please note that every set of descriptors generated two models derived from different training times (e.g., Centralities 1 refers to the best model obtained with centralities attributes during a training period of 35 h; see Methods for further details). To further test this, we performed a cross-validation (leave-one-out) test on each model and corresponding data set to estimate different (TP rate, FP rate, Precision, Recall, MCC and ROC area) statistical parameters (see Table 2, Table 3, Table 4 and Table 7). Finally, we computed these same statistical parameters to test the models obtained with JAMMING1SET using the JAMMING3SET as a swapped test and vice versa (see Table 3 and Table 8).

Our results with JAMMING1SET and 11 centralities plus one conservation score (see Table 1 and Table 2) showed that the optimum models improved on our previous method [15] aimed to predict critical residues for protein function using the same set of proteins and critical residues, but using one single centrality measure (our previous results generated precision and recall values of 0.2 and 0.8, respectively). Furthermore, using physicochemical descriptors from ProtDCal alone or combined with centralities also improved on the centralities classification of critical residues (see Table 1 and Table 2).

### 2.3. Are Critical Residues for Protein Function Best Classified Using a Unique and Quantitative Formalism?

In an attempt to further improve the classification of critical residues, we recognized that the definition of critical residues has not been standardized. For instance, in the six proteins of JAMMING1SET used to train our method to predict critical residues, each author used different criterion to define critical residues (see Materials and Methods). Hence, we hypothesize that using a common measure to define critical residues may improve their prediction. To test this idea, we implemented a method to determine the criticality of a residue based on mutagenesis data; we referred to this as the criticality index *CI* (see Materials and Methods). Briefly, *CI* takes into account the available mutants and associated phenotypes of every residue to report an index that ranks the substitutions required to achieve a wild type or a mutant phenotype. Using JAMMING2SET that annotated each residue based on a score derived from the *CI* index to identify the best models to classify critical residues for protein function we also noted that the ProtDCal descriptors rendered better classification than centralities alone (see Table 4 and Table 5). Please note that every set of descriptors generated 1 or 2 models derived from different training times (e.g., ProtDCal 1 refers to the best model obtained with ProtDCal attributes during a training period of 35 h; see Materials and Methods for further details).

### 2.4. Are Critical Residues for Protein Function Best Modeled by a Binary Classification?

We noticed that a binary definition of residues as critical or not critical seemed easier to learn (compare results between JAMMING1SET and JAMMING2SET reported in Table 1 and Table 4), hence we built JAMMING3SET to annotate residues using a binary classification derived from *CI* score (see Materials and Methods). After searching for the best models to classify critical residues for protein function based on this binary classification, we observed that the ProtDCal descriptors rendered better classification than centralities (see Table 6 and Table 7); please note that every set of descriptors generated 1 or 2 models derived from different training times (e.g., Union 1 refers to the best model obtained with centralities and ProtDCal attributes during a training period of 35 h; see Materials and Methods for further details). However, comparing these results with those obtained with the JAMMING1SET did not show large differences. The small difference may be the consequence of having a different number of instances in JAMMING1SET (1436) and JAMMING3SET (693), that is, JAMMING1SET has almost twice as many examples to learn from than JAMMING3SET. To test which classifier had better capacity to learn what a critical residue is, we performed a swapped test. For that end, we used the model identified by AutoWEKA for each set of attributes using JAMMING1SET and test this model using the JAMMING3SET (see Table 3) and vice versa (see Table 8). We observed that the only models that reached a ROC area value >0.5 (random predictors achieve up to 0.5 ROC area) were those based on the JAMMING3SET, thus our criticality index captures the variable definitions of critical residues independently used by different authors.

### 2.5. Are Critical Residues Best Classified by a Single Model?

To determine how similar each model was, we compared their classifications of critical residues (see Materials and Methods). We performed this comparison only between models trained with the same dataset that used a binary definition of critical residues. Our results show that overall there is 50% of similarities between the classifications of critical residues derived from the 4 models trained with JAMMING1SET (see Table 9) and 70% of similarities between the 4 models trained with JAMMING3SET (see Table 9). This reflects the fact that JAMMING1SET include multiple definitions of critical residues while JAMMING3SET uses only one. It is noticeable that models trained with JAMMING1SET and ProtDCal descriptors do not present any overlap with the rest of the models; alternatively, one of the models trained with JAMMING3SET and ProtDCal descriptors was redundant to the other models.

Finally, considering that critical residues are best classified following a binary classification, we implemented a filter-wrapper feature selection method to select the subset of descriptors that maximizes the classification of a Support Vector Machine (SVM). Such optimization is aimed at reducing the error in the classification of critical residues, weighting for the penalty cost C for the critical and non-critical classes in the SVM (see Materials and Methods). SVMs are known to work well in binary classifications and we wanted to test whether such algorithm, once the descriptors and parameters are optimized for critical residues classification, could recapitulate critical residues from protein structure descriptors better than the models generated by a more general optimization obtained from AutoWEKA. Our results show that the models obtained with the JAMMING1SET (see Table 2) or JAMMING3SET (see Table 7) did not improve the classifications achieved with individual models obtained from AutoWEKA, hence provided further support to the idea that critical residues classification requires the combination of multiple models.

## 3. Discussion

A systematic evaluation of different machine-learning algorithms and protein structure descriptors aimed to identify the best models to classify critical residues is reported; we aimed to achieve different goals in performing this study. On the one hand, we aimed to identify the best descriptors of protein structure to classify critical residues. Our results indicate that both ProtDCal descriptors and centralities accomplished reliable classification of critical residues. Furthermore, ProtDCal descriptors of protein structure account best for the critical role of residues than centralities. Considering that ProtDCal is a versatile library that facilitates the development of new descriptors, we expect our data and strategy may encourage the future testing of novel descriptors implemented in ProtDCal to classify critical residues for protein function.

As noted before, critical residues are those positions in a protein structure that upon mutation affect protein stability, folding and/or activity. Such definition lacks quantitative precision, for instance it does not establish how many mutations altering the stability/folding/activity of a protein are required to consider a residue as critical [22]. In an attempt to propose a solution to this limitation in the definition of critical residues, here we define a criticality index function (*CI*, see Methods); such index encompasses five different ways to account for amino acid replacements (polarity, secondary structure, molecular volume, codon diversity, and electrostatic charge) and consequently provides a quantitative estimate of how tolerant is a given position to mutants. It is important to note that previous methods have been reported to predict mutations that negatively affect protein function [23,24,25]; such approaches differ from our approach since our goal is to define which residues are critical rather than to identify which mutations may alter protein function. Our results show that *CI* is learnable by both centralities and ProtDCal descriptors, and that *CI* captures different definitions of critical residues tested in this work. A feature of our criticality index is that it does not depend on saturation mutagenesis (it works with partial mutagenesis data) to provide an estimate on the criticality of a residue. 

We also assessed the machine-learning algorithm that best classified critical residues. The current standard implementation of WEKA includes up to 55 algorithms; our results show that depending on the dataset used, different algorithms and parameters were identified to efficiently classify critical residues, hence confirming the need to evaluate in a systematic fashion the different machine-learning algorithms. For instance, support vector machine (an implementation of this algorithm in the WEKA environment is referred to as SMO) rendered the best classification using the JAMMING1SET and the union attribute set, while logistic regression improved on those results using the same dataset but different attributes (see Table 1). We also show that combining descriptors did not improve on the efficiency to classify critical residues. Our results indicate that there is on average 60% of similarity between the best 8 models identified in this study (see Table 9), suggesting that the combination of these models may render improved classifications of critical residues. However, it is important to note that because most of the residues in our datasets were annotated as non-critical (see Supplemental Files S1–S9), the statistical parameters are dominated by the classification of those residues. Despite this apparent limitation, considering all the predictions from models trained with JAMMING1SET rendered 78.6% of all critical residues annotated in that dataset and all the predictions from models trained with JAMMING3SET correctly classified 100% of known critical residues (see Supplemental Files S11–S18). These results support the notion that critical residues are indeed related to protein structure descriptors and that such relationship depends on different models, in agreement with the multiple roles critical residues have on protein function (e.g., folding, catalysis). While the goal of this work does not aim to develop an efficient meta-predictor of critical residues for protein function, these results suggest that combining multiple models to classify critical residues for protein function may render a very efficient predictor. Further studies may be required to develop such meta-predictor of critical residues.

To further test this idea, we performed an optimization to select a subset of descriptors such that the accuracy of the support vector machine is maximized. Our results indicate that even with the optimal descriptor subset the support vector machine could not fully classify critical residues (see Table 2 and Table 7). These results further support the idea that in order to obtain an efficient predictor of critical residues for protein function, it is convenient to combine different learning models. Our results may complement recent reports on protein function prediction based on machine learning methods [26,27,28,29].

## 4. Materials and Methods

All methods and their corresponding tools are supported by results in the field of machine learning, for details of these methods and references to their tools we refer to the reader to a review in the field [30].

### 4.1. Set of Proteins

The JAMMING1SET contains six proteins for which the atomic three-dimensional structure was known and included the following PDB entries used for training: 1GVP, 1ZG4, 2NPH, 2P9H, 2ZD1 and 3LZM; chain A was used for each of these proteins to derive their structure descriptors (see Supplementary Files S1–S3). The JAMMING2SET included the following PDB entries: 1BNK, 1HIV, 1ZG4, 2PR6 and 4UXZ; again, chain A was used for each of these proteins to derived their structure descriptors (see Supplementary Files S4–S6). Finally, JAMMING3SET included the following PDB entries: 1BNK, 1HIV, 1ZG4, 2PR6 and 4UXZ (see Supplementary Files S7–S9). The mutants of each residue for the JAMMING1SET and JAMMING2SET was based on the original report for each protein or derived from the reported mutations at each position: 1BTL [31], 1BNK [32], 1GVP [33], 1HIV [34], 1ZG4 [31], 2NPH [34], 2P9H [35], 2PR6 [36], 2ZD1 [37], 3LZM [38] and 4UXZ [39]. Only residues in JAMMING1SET where there was an annotation from the authors of these works about the residue being critical or not were considered in our analysis; all residues where there was uncertainty or lack of knowledge about the critical role of that position for protein function were annotated as not critical. Briefly, critical residues were defined as follows: (i) for the HIV-1 protease were residues where non-conservative mutations rendered a mutant phenotype; (ii) for the HIV-1 retrotranscriptase were those where 75% of the non-conservative mutations rendered a mutant phenotype; (iii) for the gene V of the bacteriophage f1, critical residues were those with a tolerance (T_k_) value less or equals to 0.4; (iv) for the T4 lysozyme and the Lac repressor, critical residues were those where 25% or less of the mutations rendered a wild-type phenotype and (v) for the beta-lactamase, critical residues were those where non-conservative mutations did not render a wild-type phenotype. The criticality for residues in the JAMMING2SET was derived from our criticality index (see below).

### 4.2. Protein Structure Descriptors

2789 ProtDCal descriptors at the residue level were obtained from the PDB files listed above. These descriptors were depurated using WEKA version 3.8 (University of Waikato, Hamilton, New Zealand) remove useless filter (these are descriptors that did not change among the different instances provided), which eliminated 329 of these descriptors. The project file (“.proj”) used to generate these descriptors using ProtDCal is available as Supplemental File S10.

From a PDB file, a contact map of residues is built as previously described [16]. This graph is undirected and we implemented a code that computes from such graph 11 centrality measures (see Table 10) and the correlated mutation index, see below. The calculated centrality values and correlated mutation index values for every PDB file in every dataset are available as Supplemental File S1.

### 4.3. Correlated Mutation Index of Contacting Residues 

**Figure 2:**
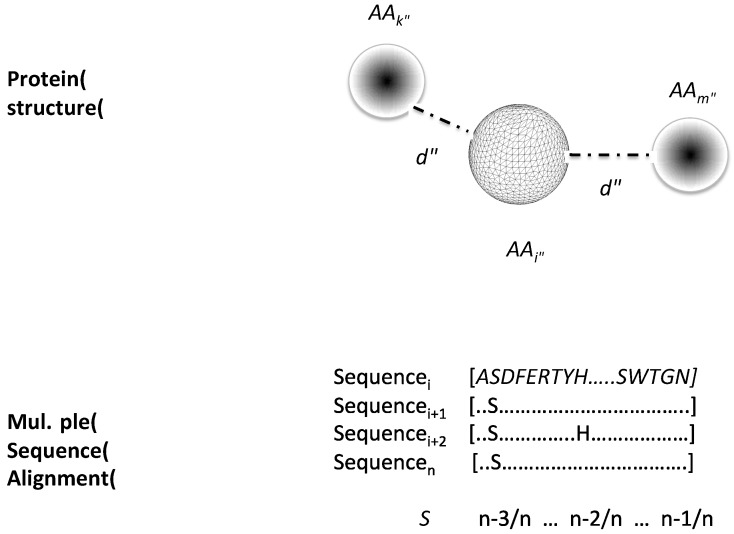
Correlated mutation index of contacting residues. The image represents in the upper part the contacting residues (*AA*_k_ and *AA*_m_) for residue *AA*_i_ in the three-dimensional protein structure; a distance (*d*) criterion of 5 Å defined contacting residues (see Materials and Methods). In the lower part, the substitution rate (*S*) is derived from a structure-based multiple sequence alignment for each of the residues in contact; the bottom part of the image shows an example on how to obtain the S parameter (for a more detailed description, please read the corresponding Methods section). Each residue in a sequence is represented by a one-letter code; only conserved residues are shown in the alignment, the rest of the sequence is presented as dots.

We developed an index to account for the correlated mutations observed in a multiple sequence alignment between residues that in the protein’s three-dimensional structure are in contact. The idea for this index is to account for positions that are relevant for protein structure. To do so, we first computed for every residue within 5 Å apart from each other. Then, for each pair of residues at 5 Å apart in the protein structure we computed the observed normalized frequency of residue substitutions (*S*); this *S* for every residue was derived from the multiple sequence alignments reported at the HSSP [40] for two sets of proteins: (i) 150 globular proteins with different folds and PFAM domains [41] and (ii) 588 transmembrane proteins at the TOPDB [42]. This normalized frequency was then used to compute the correlated mutation index (*CM*) of every *j*-residue as follows (see Figure 2 and Equation (3)):

(3) $$CM_{j}=\sum_{i=1}^{i=m}S_{i}$$

where *m* corresponds to the number of contacting residues for *j*-residue in the three-dimensional structure of a protein; that is, if a residue has *m* contacting residues (those at 5 Å in the protein structure), its *CM* corresponds to the sum of the individual substitution frequencies (*S_i_*) of every contacting residue. The calculated index values together with the centralities described above applied to the three datasets are available as Supplemental File S1.

### 4.4. Function Criticality Index from Site-Directed Mutagenesis Experiments

To standardize the criteria to define critical residues for protein function, we developed an index that depends on 5 descriptors previously described for proteins sequences [43]; hence, a vector of 5 dimensions represents each residue in a protein. The Euclidean distance then can be computed for every mutation from the original residue to the mutated one. Each mutation is ranked according to the position they occupied in the list of all possible 380 different residue’s substitutions; among the 400 possible residue mutations, 20 corresponds to substitutions that render the same residue thus are not considered as mutations. The ranking, in this case, goes in a descending order so that the most dissimilar mutations will have high ranking values, hence representing a high probability of being critical. The criticality index *CI* for a protein residue is defined as follows (Equation (4)):

(4) $$CI=\frac{\sum_{i=1}^{i=m}R_{i}}{\sum_{i=380-m}^{i=380}i}$$

where *R*_i_ corresponds to the ranking position of every one of the *m*-mutations reported for *i*-position. Thus, positions with a low *CI* value will be tolerant to mutations and positions with high *CI* value will be less tolerant to mutations. The definition of a critical residue then is defined based on the relative value of the ranking of the mutations reported for that position. As defined in the denominator of equation 3, the top *m* ranking values among the 380 different single residue mutations are used to determine the criticality of the set of *m*-ranked mutations. If a residue has mutations reported to render both wild type and mutant phenotypes, a modification on the formula is applied as follows (Equation (5)):

(5) $$CI=\frac{1}{2}*[(1-\frac{\sum_{i=1}^{i=m}RW_{i}}{\sum_{i=380-m}^{i=380}i})+\frac{\sum_{i=1}^{i=m}RM_{i}}{\sum_{i=380-m}^{i=380}i}]$$

where *RW_i_* and *RM_i_* correspond with the rankings for the wild-type and mutant phenotypes respectively. The calculated indexes for the data sets used in this study are available as Supplemental Files S4–S6.

### 4.5. Selection of Protein Structure Descriptors

Two selections were conducted in our study. The first one, RemoveUseless method in WEKA, eliminated all descriptors that did not vary among the different instances of critical residues. AutoWEKA performed the other selection of descriptors; when such selection was observed to generate the best prediction of critical residues, the selection method was reported.

### 4.6. Combined Selection and Parameter Optimization Using AutoWEKA

AutoWEKA was used in a command-line fashion to identify the best algorithm and its optimal parameters to learn from the training data set. The command line used was:

java –cp [path-to-autoweka.jar]:[path-to-weka.jar] -Djava.io.tmpdir = [path_to-save-autoweka-tmp-files] weka.classifiers.meta.AutoWEKAClassifier –t [path-to-arff-training-dataset] –d [path-to-model-output-filename] –timeLimit [1500 or 3000] -memLimit 32000 > [path-to-output-file-with-statistics]

The arff file used for training is available as supplementary material (Supplemental Files S1–S9). This command was executed on a Linux machine with 512 GB of RAM and 24 cores. The autoweka.jar and weka.jar files were downloaded from their official distribution sites.

### 4.7. Comparison of Critical Residue Classification among Different Models

For every model trained to classify critical residues we compared their classifications to determine how similar these were. To do so, we identified every residue classified as critical for each model and that number was used to normalize the number of identical classifications between each pair of models. The predictions as critical residues for each model and test set are available as Supplemental Files S11–S18.

### 4.8. A Filter-Wrapper Method for Selecting Descriptors in the Critical Residues Classification Problem

We implemented a two-stage feature selection procedure that uses a filter in the first stage and a wrapper at the second stage. The filter method was employed to remove redundant and irrelevant descriptors for each descriptor set. For the remainder descriptors, a wrapper method was used to determine the subset of descriptors that improve the performance for critical residue classification. The main details of the feature selection method are described below.

#### 4.8.1. Filter Method

The Spearman’s correlation coefficient and the information gain were used to evaluate the redundancy and irrelevancy of each descriptor, respectively. To remove highly correlated descriptors from the dataset we used in-house remove Correlated Attributes program (available as Supplemental File S19 and at https://github.com/3eltran/RemoveCorrelatedAttributes). The command line used was: 

Python3.6 (path-to removeCorrelatedAttributes.py) -i (path-to-arff-training-dataset) -o (path-to-output-filename).

WEKA filter attribute selection was used to remove descriptors with information gain values equal to zero. The used command line was: 

java -cp [path-to-weka.jar] weka.filters.supervised.attribute.AttributeSelection -E “weka.attributeSelection.InfoGainAttributeEval” -S “weka.attributeSelection.Ranker -T 0 -N -1” -i [path-to-arff-training-dataset] -o [path-to-output-filename].

Training sets to be used in this command were derived from the removeCorrelatedAttributes.py program (Supplemental File S19) and are available as Supplemental Files S20–S25.

#### 4.8.2. Wrapper Method

To determine the subset of descriptors that improves classification accuracy, we implemented a wrapper method. It consists of two modules: a search strategy, and an evaluation measure.

Search strategy. In practice, searching over all possible subset of descriptors is not feasible because the size of the search space for *m* descriptors is 2^m^ − 1. For this reason, we implemented a genetic algorithm to explore efficiently the search space. Genetic Algorithms (GAs) are known to work well in large-scale problems with more than 50 descriptors, furthermore GAs are capable of avoiding getting stuck at local optima [44]. The main components of the implemented GA are described next. 

First, a chromosome was constructed with a subset of indices, where each index corresponds to a selected descriptor. The initial population consists of 50 randomly generated chromosomes with a size between 1 and (m/10), m denotes the number of descriptors. Afterwards we employed a binary tournament with replacement as parent selection scheme. For each pair of parents, we applied the Subset Size-Oriented Common Feature (SSOCF) [45] crossover operator with a probability of 0.7. Then, we applied the mutation operator with probability (pm) equal to 0.1. This operator consists in generating a random index according to the uniform distribution between 0 and m. If the generated index is within the chromosome then it is removed, otherwise it is added to the chromosome. To select the chromosomes that will form part of the next generation, we used elitism.

In the evaluation measure module, the quality of each chromosome was evaluated. First, the chromosome was decoded into a dataset with only the selected features. Next, the dataset was employed as an input to the support vector machine (SVM) in order to generate the classification model. The SVM was used from WEKA where it is referred to as LIbLINEAR. Considering the class imbalance in JAMMING1SET and JAMMING3SET, we applied regularization to rescale the penalty cost C for the critical and non-critical classes. The weights employed for the JAMMING1SET were 9 and 3 for the critical and non-critical classes, respectively. Alternatively, the weights for the JAMMING3SET were 5 and 1.22 for the critical and non-critical classes. In order to evaluate the generated model, we performed a 10-fold cross-validation and the Matthew’s Correlation Coefficient (MCC) was used as a performance measure to select the best subset of descriptors.

## 5. Conclusions

In this work a systematic strategy to test descriptors and machine-learning algorithms to classify critical residues for protein function is presented. Also, a mathematical criterion to represent mutagenesis experiments is introduced to quantify the criticality of a residue for protein function. Our results show that our function criticality index recapitulates different definitions of critical residues and provide evidence that physicochemical and centralities descriptors of protein structure effectively classify critical residues. Our results provide the basis to further improve on the development of models to predict critical residues.

## Figures and Tables

**Table 1:** Algorithms and parameters optimized for JAMMING1SET.

Descriptors	Algorithm	Parameters	Relative Absolute Error (%)
Centralities 1	Multilayer Perceptron	[-L, 0.5571819547734898, -M, 0.23520521436874284, -B, -H, o, -C, -D, -S, 1] Attribute search: GreedyStepwise [-C, -B, -R] Attribute evaluation: CfsSubsetEval [-L]	77.2
Centralities 2	LWL	[-A, weka.core.neighboursearch.LinearNNSearch, -W, weka.classifiers.functions.MultilayerPerceptron, --, -L, 0.3899191912662868, -M, 0.4683563849238558, -H, o, -C, -R, -D, -S, 1]	78.2
ProtDCal 1	Logistic	[-R, 0.057140274761388915] Attribute search: BestFirst [-D, 1, -N, 4] Attribute evaluation: CfsSubsetEval [-L]	67.9
ProtDCal 2	Logistic	[-R, 0.057140274761388915] Attribute search: BestFirst [-D, 1, -N, 4] Attribute evaluation: CfsSubsetEval [-L]	67.9
Union 1	SMO	[-C, 0.7127949742291734, -N, 0, -M, -K, weka.classifiers.functions.supportVector.PolyKernel -E 1.1133446901320447 -L] Attribute search: GreedyStepwise [-C, -N, 898] Attribute evaluation: CfsSubsetEval [-L]	68.6
Union 2	SMO	[-C, 0.7127949742291734, -N, 0, -M, -K, weka.classifiers.functions.supportVector.PolyKernel -E 1.1133446901320447 -L] Attribute search: GreedyStepwise [-C, -N, 898] Attribute evaluation: CfsSubsetEval [-L]	68.6

Three sets of descriptors (Centralities, ProtDCal and union, see Materials and Methods) used to identify the best algorithm and corresponding parameters to learn the annotated critical and non-critical residues from the data set referred to as JAMMING1SET (see Materials and Methods). For each set of descriptors, AutoWEKA was executed at two different duration times: 1500 (model 1) and 3000 (model 2) min. We report these two results to show that no significant improvement was observed by doubling the executing time of AutoWEKA. AutoWEKA may perform the selection of descriptors; when such selection rendered the best model, this is specified (Attribute search, Attribute evaluation).

**Table 2:** Leave-One-Out Test for JAMMING1SET.

Attribute Set	TP Rate	FP Rate	Precision	Recall	MCC	ROC Area
Centralities 1	0.83	0.53	0.81	0.83	0.37	0.77
Centralities 2	0.83	0.51	0.81	0.83	0.37	0.79
ProtDCal 1, 2	0.84	0.52	0.82	0.84	0.39	0.81
Union 1, 2	0.84	0.53	0.82	0.84	0.38	0.81
SVM-Centralities	0.79	0.33	0.82	0.79	0.41	0.73
SVM-ProtDCal	0.81	0.27	0.84	0.81	0.48	0.81
SVM-Union	0.82	0.25	0.85	0.82	0.49	0.78

Evaluation of different models trained with JAMMING1SET using the leave-one-out test. The reported statistical parameters are the weighted averages, as reported by WEKA. The attribute sets (Centralities, ProtDCal and Union) are divided into one or two groups, corresponding to the models obtained at 1500 or 3000 min of optimization performed by AutoWEKA (see Table 1). The last rows labeled with SVM- show the results obtained with the filter-wrapper feature selection method based on the support vector machine implementation in WEKA referred to as LibLINEAL using different descriptors (see Materials and Methods).

**Table 3:** Swapped Test for JAMMING1SET Best Models.

Attribute Set	TP Rate	FP Rate	Precision	Recall	MCC	ROC Area
Centralities 1	0.61	0.67	0.55	0.61	−0.07	0.42
ProtDCal 1	0.58	0.60	0.54	0.58	−0.02	0.45
Union 1	0.58	0.61	0.74	0.58	−0.02	0.40

The JAMMING3SET was used to test the best models trained with JAMMING1SET. The reported statistical parameters are the weighted averages, as reported by WEKA. The attribute set names (Centralities, ProtDCal and Union) are followed by a number 1, corresponding with the models obtained at 1500 min of optimization performed by AutoWEKA (see Table 1).

**Table 4:** Algorithms and parameters optimized for JAMMING2SET.

Descriptors	Algorithm	Parameters	Relative Absolute Error (%)
Centralities 1	Linear Regression	[-S, 2, -R, 7.855468822045874E-7], Attribute search: GreedyStepwise [-C, -B, -N, 172], Attribute evaluation: CfsSubsetEval []	82.8
Centralities 2	LWL	[-A, weka.core.neighboursearch.LinearNNSearch, -W, weka.classifiers.functions.LinearRegression, --, -S, 0, -R, 0.20912016083576357], Attribute search: GreedyStepwise [-C, -N, 213], Attribute evaluation: CfsSubsetEval [-L]	82.4
ProtDCal 1	M5P	[-M, 1, -R]	80.1
ProtDCal 2	M5P	[-M, 1, -R]	80.1
Union 1	Bagging	[-P, 74, -I, 8, -S, 1, -W, weka.classifiers.trees.DecisionStump, --]	88.5
Union 2	Bagging	[-P, 74, -I, 8, -S, 1, -W, weka.classifiers.trees.DecisionStump, --]	88.5

Three sets of descriptors (Centralities, ProtDCal and union, see Materials and Methods) used to identify the best algorithm and corresponding parameters to learn the annotated critical and non-critical residues from the data set referred to as JAMMING2SET (see Materials and Methods). For each set of descriptors, AutoWEKA was executed at two different duration times: 1500 (model 1) and 3000 (model 2) min. We report these two results to show that no significant improvement was observed by doubling the executing time of AutoWEKA. AutoWEKA may perform the selection of descriptors; when such selection rendered the best model, this is specified (Attribute search, Attribute evaluation).

**Table 5:** Leave-One-Out Test for JAMMING2SET.

Attribute Set	Correlation Coefficient	Relative Absolute Error (%)
Centralities 1	0.54	83.2
Centralities 2	0.55	83.0
ProtDCal 1, 2	0.64	74.5
Union 1, 2	0.52	83.7

Evaluation of different models trained with JAMMING2SET using the leave-one-out test. The reported statistical parameters correspond with the cross-validation results, as reported by WEKA. The attribute sets (Centralities, ProtDCal and Union) are divided into 1 or 2 groups, corresponding with the models obtained at 1500 or 3000 min of optimization performed by AutoWEKA (see Table 4).

**Table 6:** Algorithms and parameters optimized for JAMMING3SET.

Descriptors	Algorithm	Parameters	Relative Absolute Error (%)
Centralities 1	IBk	[-K, 13]	82.3
Centralities 2	AdaBoostM1	[-P, 79, -I, 108, -Q, -S, 1, -W, WEKA.classifiers.functions. MultilayerPerceptron, -L, 0.7665662779502016, -M, 0.21535709618934423, -B, -H, i, -C, -R, -D, -S, 1]	99.8
ProtDCal 1	Naïve Bayes	[-D] Attribute search: GreedyStepwise [-C, -N, 161], Attribute evaluation: CfsSubsetEval [-L]	61.5
ProtDCal 2	J48	[-J, -A, -S, -M, 18, -C, 0.3184313887632543] Attribute search: BestFirst [-D, 1, -N, 7] Attribute evaluation: CfsSubsetEval [-M]	67.5
Union 1	Simple Logistic	[-W, 0]	100
Union 2	J48	[-J, -A, -S, -M, 18, -C, 0.3184313887632543] Attribute search: BestFirst [-D, 1, -N, 7] Attribute evaluation: CfsSubsetEval [-M]	67.5

Three sets of descriptors (Centralities, ProtDCal and Union, see Materials and Methods) were used to identify the best algorithm and corresponding parameters to learn the annotated critical and non-critical residues from the JAMMING3SET. For each set of descriptors, AutoWEKA was executed at two different duration times: 1500 (model 1) and 3000 (model 2) min. We report these two results to show that no significant improvement was observed by doubling the execution time of AutoWEKA. AutoWEKA may perform the selection of descriptors; when such selection rendered the best model, this is specified (Attribute search/evaluation).

**Table 7:** Leave-One-Out Test for JAMMING3SET.

Attribute Set	TP Rate	FP Rate	Precision	Recall	MCC	ROC Area
Centralities 1	0.76	0.50	0.75	0.76	0.34	0.76
ProtDCal 1	0.83	0.61	0.80	0.83	0.31	0.79
ProtDCal 2	0.82	0.55	0.80	0.82	0.32	0.68
Union 1	0.84	0.55	0.82	0.84	0.37	0.81
Union 2	0.84	0.49	0.82	0.84	0.41	0.73
SVM-Centralities	0.72	0.26	0.77	0.72	0.42	0.72
SVM-ProtDCal	0.74	0.29	0.76	0.74	0.42	0.72
SVM-Union	0.74	0.19	0.81	0.74	0.50	0.77

Evaluation of different models trained with JAMMING1SET using the leave-one-out test. The reported statistical parameters are the weighted averages, as reported by WEKA. The attribute sets (Centralities, ProtDCal and Union) are divided into 1 or 2 groups, corresponding with the models obtained at 1500 or 3000 min of optimization performed by AutoWEKA (see Table 6). The last rows labeled with SVM- show the results obtained with the filter-wrapper feature selection method based on the support vector machine implementation in WEKA referred to as LibLINEAR using different descriptors (see Materials and Methods).

**Table 8:** Swapped Test for JAMMING3SET Best Models.

Attribute Set	TP Rate	FP Rate	Precision	Recall	MCC	ROC Area
Centralities 1	0.54	0.78	0.63	0.54	−0.2	0.30
ProtDCal 2	0.72	0.76	0.68	0.72	−0.04	0.53
Union 2	0.72	0.76	0.68	0.72	−0.04	0.53

The JAMMING1SET was used to test the best models identified using the JAMMING3SET. The reported statistical parameters are the weighted averages, as reported by WEKA. The attribute sets (Centralities, ProtDCal and Union) are divided into 1 or 2 groups, corresponding with the models obtained at 1500 or 3000 min of optimization performed by AutoWEKA (see Table 7).

**Table 9:** Comparison of classifications between critical residues models.

	Training Set	Centralities 1	Centralities 2	ProtDCal 1, 2	Union 1, 2	Average
Centralities 1	1	100	78	0	62	60
Centralities 2	1	92	100	0	63	63
ProtDCal 1, 2	1	0	0	100	0	25
Union 1, 2	1	66	56	0	100	55
Centralities 1	3	100	47	50	65	65
ProtDCal 1	3	35	100	100	84	79
ProtDCal 2	3	28	75	100	69	68
Union 2	3	37	63	69	100	67

Different models trained with JAMMING1SET (Training set column indicated by number 1) or JAMMING3SET (Training set column indicated by number 3) were compared in their classifications of critical residues. The table shows the percentage of identical critical residues achieved in such comparison. Comparisons were performed only where critical residues were treated as binary and the descriptors and instances were identical. Note that the table is asymmetrical as a consequence that the percentages are reported based on the number of critical residues classifications obtained from each model indicated in each row. For instance, when the training set was 3 (JAMMING3SET) the classifications of critical residues from model trained on ProtDCal 1 are 100% identical to those obtained from ProtDCal 2, yet ProtDCal 2 is only 75% identical to ProtDCal 1; this indicates that there are more classifications of critical residues derived from ProtDCal 2 than from ProtDCal 1 and that all the classifications from ProtDCal 1 are included in the classifications derived from ProtDCal 2 (see Materials and Methods). Models obtained with Centralities at 3000 min or Union at 1500 min were not analyzed because these rendered large errors (see Table 6).

**Table 10:** Centrality measures used in this study.

Centrality	Description
Excentricity	This is defined as the longest shortest distance of a node. The shortest distance was computed using the Dijkstra’s algorithm.
Excentricity inverted	1/Excentricity
Degree	This is defined as the number of contacts for any residue in the graph.
Sphere degree	For any given i-residue, identify its neighbors and count the number of contacts. This is the degree at a second level of i-node.
Sphere degree accumulated (SNN)	Is derived as the sphere degree, but the counts include the number of neighbors of i-residue.
Mean distance	This is the sum of the shortest distances recorded from i-residue to any other residue divided by the number of neighbors for i-residue.
Closeness centrality	Is derived from the calculation of the shortest distances, according to the Dijkstra’s algorithm, between i-residue and the other residues in the protein. Closeness is the inverse of the sum of all these distances and is equivalent to 1/mean distance.
Clustering coefficient	Is obtained by dividing the observed number of neighbors between the neighbors of i-residue (o) by the expected number of neighbors (n): o/(n*(n − 1)).
Clustering coefficient inverted	1/Clustering coefficient
Traversity	This index measures the number of times a residue is traversed while connecting every pair of residues in the contact map using the shortest path from the Dijkstra’s algorithm. Two version of this centrality are produced: One that follows the order of residues in the protein sequence (Traversity A) and another that does not (Traversity B).

## Supplementary Materials

Supplementary materials are available online. File S1: Training set JAMMING1SET with centralities descriptors, File S2: Training set JAMMING1SET with ProtDCal descriptors, File S3: Training set JAMMING1SET with the union descriptors, File S4: Training set JAMMING2SET with centralities descriptors, File S5: Training set JAMMIN2SET with ProtDCal descriptors, File S6: Training set JAMMING2SET with the union descriptors, File S7: Training set JAMMING3SET with centralities descriptors, File S8: Training set JAMMING3SET with ProtDCal descriptors, File S9: Training set JAMMING3SET with the union descriptors, File S10: ProtDCal project file, File S11: Predictions of critical residues achieved with model 1 trained with JAMMING1SET and centralities descriptors, File S12: Predictions of critical residues achieved with model 2 trained with JAMMING1SET and centralities descriptors, File S13: Predictions of critical residues achieved with model 1/2 trained with JAMMING1SET and ProtDCal descriptors, File S14: Predictions of critical residues achieved with model 1/2 trained with JAMMING1SET and the union descriptors, File S15: Predictions of critical residues achieved with model 1 trained with JAMMING3SET and centralities descriptors, File S16: Predictions of critical residues achieved with model 1 trained with JAMMING3SET and ProtDCal descriptors, File S17: Predictions of critical residues achieved with model 2 trained with JAMMING3SET and ProtDCal descriptors, File S18: Predictions of critical residues achieved with model 2 trained with JAMMING3SET and the union descriptors, File S19: Remove Correlated Attributes program, File S20: ARFF with selected attributes from JAMMING1SET and centralities using S19, S21: ARFF with selected attributes from JAMMING1SET and ProtDCal using S19, S22: ARFF with selected attributes from JAMMING1SET and union using S19, S23: ARFF with selected attributes from JAMMING3SET and centralities using S19, S24: ARFF with selected attributes from JAMMING3SET and ProtDCal using S19 and S25: ARFF with selected attributes from JAMMING3SET and union using S19.
